# Pelvic Ewing sarcoma: a retrospective outcome analysis of 104 patients who underwent pelvic tumor resection at a single supra-regional center

**DOI:** 10.1186/s13018-020-02028-3

**Published:** 2020-11-16

**Authors:** Wiebke K. Guder, Jendrik Hardes, Markus Nottrott, Anne Juliane Steffen, Uta Dirksen, Arne Streitbürger

**Affiliations:** 1 Department of Orthopedics and Tumor Orthopedics, University Hospital Muenster, Albert-Schweitzer-Campus 1, Building A1, 48149 Muenster, Germany; 2 Department of Orthopedic Oncology, University Hospital Essen, Hufelandstrasse 55, 45147 Essen, Germany; 3 Department of Pediatric Hematology and Oncology (III), University Hospital Essen, Hufelandstrasse 55, 45147 Essen, Germany

**Keywords:** Ewing sarcoma, Pelvis, Pelvic tumor resection, Internal hemipelvectomy, Hindquarter amputation, Radiation

## Abstract

**Background:**

Local treatment in pelvic Ewing sarcoma (ES) consists of operation, radiation therapy, or a combination of both. Reported outcomes vary depending on the treatment modality performed. It is the objective of this study to analyze surgical outcome and complications as well as oncological outcome and complications of chemo- and radiation therapy in this patient cohort and evaluate prognostic factors.

**Methods:**

Retrospective review of 104 patients who underwent tumor resection for pelvic ES from 1988 to 2014.

**Results:**

All patients underwent pelvic resection and radiation therapy was administered in 77.9%. Margins were clear in 94.2%. The response to chemotherapy was good in 78.8%. Local recurrence occurred in 7.7%. The presence of distant metastases at the time of operation was the most important negative predictor for overall survival (*p* = 0.003). The cumulative 5- and 10-year survival rates were 82.7% and 80.1% for non-metastasized and 61.4% and 41.6% for metastasized pelvic ES at operation. In the presence of a single-distant metastatic site at operation compared to multiple metastatic sites, the cumulative survival rates were 64.3% versus 50% at five and 50.7% versus 16.7% at 10 years.

**Conclusions:**

A combined treatment approach of tumor resection and radiation therapy leads to a local control and overall survival rates comparable with those of extremity locations in this study’s patient cohort with localized pelvic ES. Therefore, surgical tumor resection (combined with (neo-)adjuvant radiation therapy) in non-metastatic pelvic ES seems feasible. In metastatic patients, however, the significance of tumor resection as a part of local treatment remains less certain and improved outcomes of combined local treatment approaches need to be weighed against these patients’ prognosis and quality of life.

## Background

Pelvic Ewing sarcoma (ES) accounts for roughly a quarter of all primary tumor sites with literature reports ranging between 15 and 35% [1–9]. Since 1970, the introduction of chemotherapy drastically improved the overall prognosis of ES but survival of pelvic primaries remained inferior to that of extremity locations [6, 7, 10–13]. Studies reported in the 1980s were mostly concerned with analyzing different multi-agent chemotherapy combinations, the sequence of local treatment, and evaluation of outcomes [14, 15]. Local treatment most often consisted of radiation therapy alone [14, 16, 17], but tumor resection gained more attention as a local treatment modality towards the end of that decade [13, 18–21]. Surgical treatments reported were diverse, including biopsies only, exploratory surgeries, and incomplete as well as complete resections [13]. In addition, the decision to pursue any kind of tumor resection was not a main subject of investigation and left to the primary investigator [15]. Most studies investigated pelvic primaries as part of a larger study cohort including other axial and extremity locations [9, 14–19, 21–23]. Only Evans et al. investigated sixty-two pelvic primaries and Thomas et al. seven pelvic primaries without including other primary sites [13, 20]. Despite their differences in study design, all authors agreed that pelvic primaries had the least favorable prognosis compared with all other sites, tended to relapse sooner, and had a higher rate of local relapse and lower disease-free and overall survival rates [14, 15, 19–21, 24]. With regard to treatment modalities, they reported that radiation therapy alone did not consistently achieve permanent local control and tumor resection showed a trend towards better survival rates [18]. Yet, while Wilkins et al. proposed resection of ES primaries as part of a multimodal treatment concept including chemotherapy and optionally additional radiation therapy, the significance of tumor resections for pelvic primaries remained unclear [19, 23]. Pelvic ES’s infamous and dismal prognosis led to an increased effort of analyzing outcomes in treated patient cohorts since the 1990s. Some studies did not find differences in disease-free and overall survival by comparing operatively treated pelvic ES with or without radiation therapy with radiation therapy alone [8, 10, 12]. Meanwhile, other studies published improved local control and overall survival rates for patients who underwent pelvic tumor resection or combined local treatment [2, 11, 25–30]. In 2016, Foulon et al. reported that even patients with complete tumor necrosis after neoadjuvant chemotherapy had a significant benefit from postoperative radiation therapy in their study [31]. Whelan et al. also published unexpected survival differences observed in a joint clinical trial, EICESS-92. Those significant differences of a 5-year event-free survival (EFS; 43% and 57%) and 5-year overall survival (OS; 53% and 66%) were caused because the patients of the United Kingdom (UK) study group were less likely of having been treated by both surgery and radiotherapy (18 vs. 59%). Instead, they were more likely treated using a single local therapy modality (72 vs. 35%) [32]. Andreou et al. reported that their Euro-EWING 1999 trial analysis suggested that a combined surgical and radiation approach appeared to be associated with a higher overall survival in pelvic Ewing sarcoma [33].

Among risk factors leading to worse event-free and overall survival in pelvic ES, larger tumor size, elevated local recurrence rates, and a higher rate of distant metastases at diagnosis were identified [1, 11, 34]. The inherent risks of pelvic resection associated with an elevated rate of permanent physical disability and long-term complications of operation, chemotherapy, and radiation treatment [1, 35] complicate patient counseling, and a lively debate remains with regard to choice of local treatment modalities depending also on stage of disease.

Therefore, it is the purpose of this study to analyze surgical outcome and complications as well as oncological outcome and complications of both chemo- and radiation therapy in this collective of 104 patients treated by pelvic tumor resection in 100% and additional radiation therapy in 77.9%. We also seek to identify prognostic factors observed in this study cohort.

## Materials and methods

A retrospective review of ES patients treated by pelvic tumor resection at a single supra-regional center from 1988 to 2014 was performed. All patients (*n* = 104) included in this study were chosen from a surgical database. Patient, tumor, treatment, survival, and relapse-associated data were acquired from orthopedic patient records and treatment files as well as primary source data collected in the German Society for Pediatric Oncology and Hematology (GPOH) Ewing’s sarcoma database for registered patients. If patients did not follow-up in the outpatient clinic, patients, family members, attending oncologists or local physicians, and the local registration office were contacted for follow-up information. Collection of follow-up data continued until October 2016, leading to a follow-up of at least 2 years after pelvic tumor resection in all but two patients with a follow-up of 22 and 23 months, respectively. Patients who were counseled to undergo definitive radiation therapy or decided against tumor resection did not follow-up in our department.

Prior to treatment initiation, ES was confirmed by histological examination of bioptic tissue gained from the pelvic primary. Diagnosis of pelvic ES was ascertained by both fluorescence-in-situ hybridization and analysis of EWSR1 translocation status (see Table 1). Chemotherapy was then administered according to the CESS86, (EI)CESS92, Euro-EWING 1999, and 2008 trial protocols depending on the time of diagnosis.

**Table 1:** Patient and treatment characteristics

	All patients		Primary		Bone metastasis		Multifocal		Locally recurrent		Extraskeletal		LTFU	
	***n***	%	***n***	%	***n***	%	***n***	%	***n***	%	***n***	%	***n***	%
	104	100	84	80.7	5^1^	4.8	6^2^	5.8	2^2^	1.9	2	1.9	7^1^	6.7
**Sex**														
Male	59	56.7	50	59.5	3	60	3	50	0		1	50	2	28.6
Female	45	43.2	34	40.5	2^1^	40	3^2^	50	2^2^	100	1	50	5^1^	71.4
**Age (years)**														
Mean	18.1		17.5		17.8		29		19		18.5		15.7	
Range	2–53		2–53		12–26		12–45		12–24		15–22		9–26	
**Tumor size**														
Mean (cm)	8.8													
< 9 cm	51	49	38	45.2	4^1^	80	2^2^	33.3	2^2^	100	2	100	5^1^	71.4
> 9 cm	43	41.3	38	45.2	1	20	3	50	0		0		1	14.3
Unknown	10	9.6	8	9.5	0		1	16.6	0		0		1	14.3
**Tumor volume**														
< 200 ml	18	17.3												
> 200 ml	35	33.6												
Unknown	51	49												
**Tumor location**														
Upper posterior	52	50	43	51.2	5^1^	100	2	33.3	0		0		3^1^	42.9
Lower anterior	31	29.8	25	29.8	0		2^2^	33.3	1^2^	50	0		4	57.1
Periacetabular	18	17.3	15	17.8	0		2	33.3	1	50	0		0	
Gluteal	2	1.9	0		0		0		0		2	100	0	
Unknown	1	0.9	1	1.2	0		0		0		0		0	
**Surgical procedure**														
Internal intraarticular	51	49	43	51.2	0		2^2^	33.3	1^2^	50	1	50	5	71.4
Internal extraarticular	13	12.5	9	10.7	0		2	33.3	1	50	1	50	0	
Internal (without joint involvement)	39	37.5	31	36.9	5^1^	100	2	33.3	0		0		2^1^	28.6
Hindquarter amputation	1	0.9	1	1.2	0		0		0		0		0	
**Resection type (Enneking)**														
P1a	3	2.9	0		3^1^	60	0		0		0		1^1^	14.3
P1b	1	0.9	0		1	20	0		0		0		0	
P1-2	2	1.9	2	2.4	0		0		0		0		0	
P1c	31	29.8	27	32.1	1	20	2	33.3	0		0		1	14.3
P1c + HS	2	1.9	2	2.4	0		0		0		0		0	
P1-2-3	2	1.9	2	2.4	0		0		0		0		0	
P1-2-4	14	13.5	11	13.1	0		0		0		2	100	1	14.3
P1-2-4 + HS	2	1.9	2	2.4	0		0		0		0		0	
P1-2-3-4	15	14.4	12	14.3	0		2	33.3	1	50	0		0	
P1-2-3-4 + HS	1	0.9	1	1.2	0		0		0		0		0	
P2	1	0.9	0		0		0		0		0		1	14.3
P2-3	14	13.5	12	14.3	0		1^2^	16.6	1^2^	50	0		1	14.3
P3	15	14.4	12	14.3	0		1	16.6	0		0		2	28.6
Unknown	1	0.9	1	1.2	0		0		0		0		0	
**Reconstruction types**														
Hip transposition	33	31.7												
Hip transposition w/proximal femur replacement	9	8.7												
Hip transposition spacer	2	1.9												
Pelvic implant	1	0.9	Combined with hip transposition, *n* = 1											
Flail hip	1	0.9	Combined with hip transposition, *n* = 1											
Screw-rod reconstruction w/bone cement sheath	19	18.3												
Autologous iliac bone graft osteosynthesis	6	5.8	Combined with hip transposition, *n* = 1											
Autologous fibula bone graft osteosynthesis	5	4.8	Combined with hip transposition, *n* = 1											
Allograft osteosynthesis	7	6.7												
Soft tissue reconstruction only	21	20.2												
**EWSR 1 translocation status**														
Present	62	59.6												
Absent	2	1.9												
Unknown	40	38.5												
**Histologic response to neoadjuvant chemotherapy (Salzer-Kuntschik)**														
1 (no vital tumor)	54	51.9	46	54.8	5^1^	100	1	16.6	0		0		3^1^	42.9
2 (isolated cells)	6	5.8	3	3.6	0		1	16.6	0		0		2	28.6
3 (< 10% viable cells)	22	21.2	18	21.4	0		0		0		2	100	2	28.6
4 (10–50% viable cells)	13	12.5	11	13.1	0		2^2^	33.3	1^2^	50	0		0	
5 (> 50% viable cells)	5	4.8	3	3.6	0		2	33.3	0		0		0	
6 (no response to chemo)	1	0.9	0		0		0		1	50	0		0	
Unknown	3	2.9	3	3.6	0		0		0		0		0	
**Surgical margins**														
R0	98	94.2	78	92.8	5^1^	100	6^2^	100	2^2^	100	2	100	7^1^	100
Planned R1	2	1.9	2	2.4	0		0		0		0		0	
Unplanned R1	2	1.9	2	2.4	0		0		0		0		0	
Unknown	2	1.9	2	2.4	0		0		0		0		0	
**Disease extent**														
Localized	34	32.7	34	40.5	0		0		0		0		0	
Metastatic at diagnosis	45	43.3	30	35.7	5^1,3^	100	6^2^	100	2^2^	100	2	100	2^1^	28.6
Metastatic after pelvic resection	17	16.3	17	20.2	0		0		0		0		0	
Unknown	8	7.7	3	3.6	0		0		0		0		5	71.4
**Number of metastatic organ sites**														
Single (one other organ system)			22											
Multiple (> 1 other organ systems)			7											
**Local recurrence**														
	8	7.7	6	7.1	0		2	33.3	0		0		0	
**Radiation**														
Yes	81	77.8												
Neoadjuvant	17	16.3	14	16.6	0		2^2,4^	33.3	2^2^	100	0		0	
Adjuvant	53	51	46	54.8	3	60	1	16.6	0		1	50	2	28.6
Other	11	10.6	9	10.7	0		0		0		1	50	1	14.3
No	17	16.3	12	14.3	2^1^	40	3	50	0		0		1^1^	14.3
Unknown	6	5.8	3	3.6	0		0		0		0		3	42.9

^1^One patient with pelvic bone metastasis was also lost to follow-up

^2^One patient with locally recurrent ES after definite radiation and initially multifocal ES

^3^One patient presented with a metachronic solitary bone metastasis, received pelvic resection, and developed other distant metastases later on; four other patients presented with synchronous solitary pelvic bone metastasis

^4^Both locally recurrent patients received prior definite radiation

Recommendations including local treatment modalities were discussed and approved by an interdisciplinary tumor board (ITB) with a specialty in sarcoma treatment. Recommendation for pelvic tumor resection generally required resectability of the pelvic primary with clear margins and a curative treatment intent.

Operations were carried out by seven senior orthopedic surgeons with a subspecialty in Orthopedic Oncology.

**Fig. 1:**
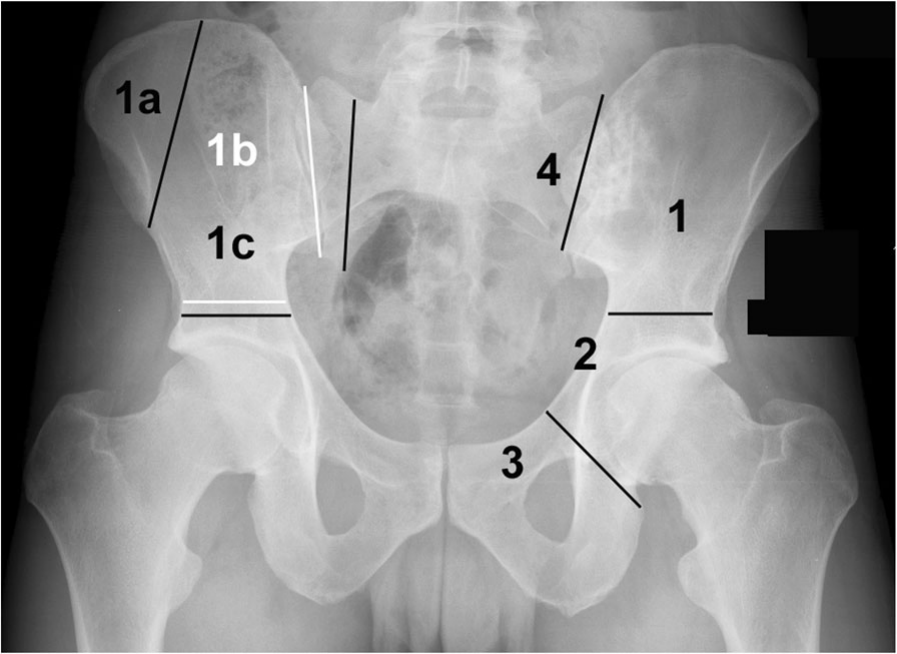
Classification of pelvic segments and resection types according to Enneking and Dunham

Tumor resections were planned with regard to tumor dimensions and reported according to the classification introduced by Enneking [36]. This classification proposed a subdivision of the hemipelvis into three areas. Iliac resections were defined as type I, acetabular resections as type II, and resections of the pubic and ischial bone as type III. Resections involving the ipsilateral sacral ala were added as type IV. Partial resections of the iliac wing leaving the pelvic ring intact were defined as type Ia, isolated type I resections as type Ib, and a resection with supraacetabular and sacral ala osteotomies with resection of the sacroiliac joint (type I and type IV) as type Ic resections. Partial or combined resections of these defined areas were frequently indicated. “Type” was substituted for “P” in this study. Pelvic resection types and areas are illustrated in Fig. 1.

**Fig. 2:**
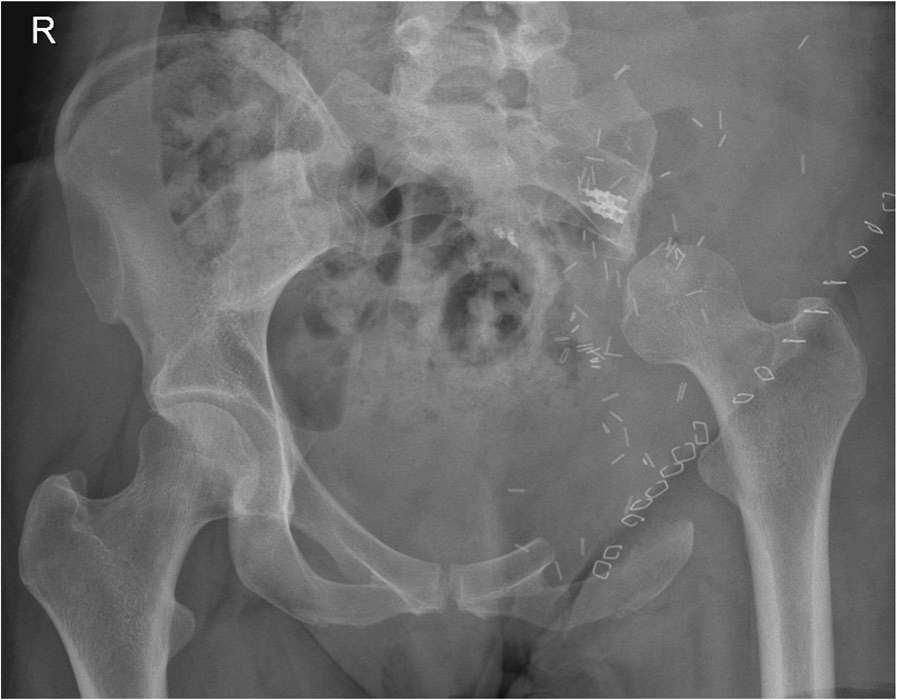
Plain radiograph of a pelvis a.p. after intraarticular, internal hemipelvectomy (types 1–2 and partially 3) and hip transposition. Two screw anchors were implanted in the sacrum for suspension of the femoral head inside an attachment tube

**Fig. 3:**
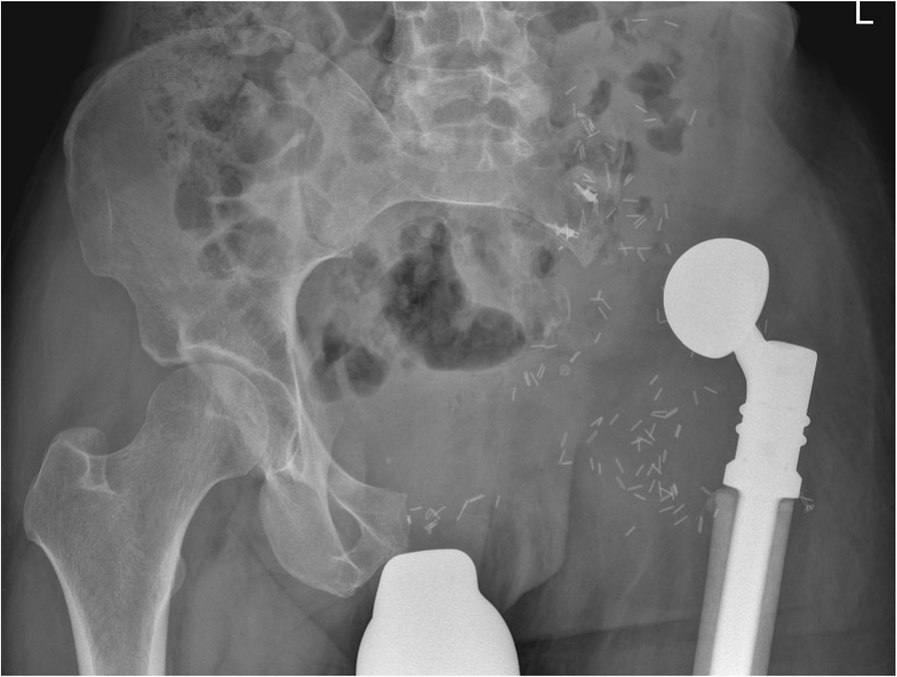
Plain radiograph of a pelvis a.p. after extraarticular, internal hemipelvectomy (types 1–3) and hip transposition. Vessel clips mark the extent of tumor resection prior to reconstruction

**Fig. 4:**
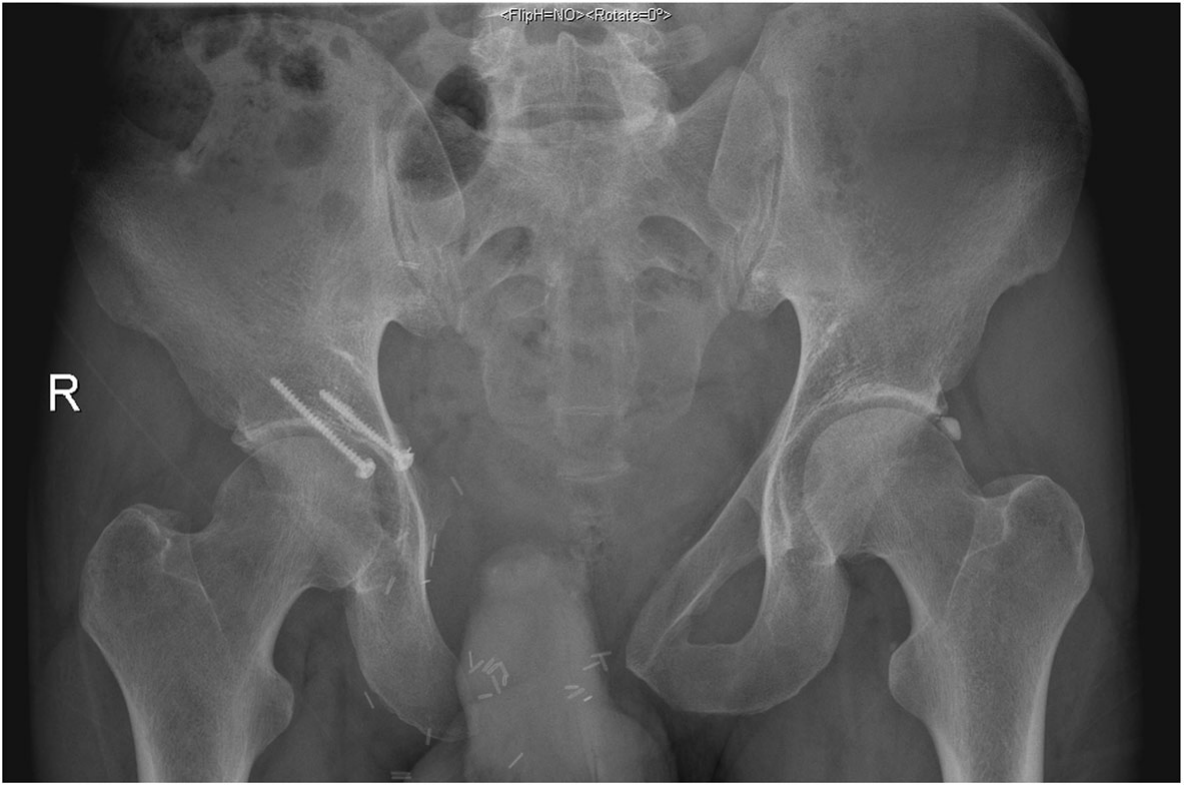
Plain radiograph of a pelvis a.p. after partial type 3 resection of the pubic bone. A partial acetabular defect was stabilized using an autologous iliac crest graft osteosynthesis. The donor site is visible on the right iliac crest

**Fig. 5:**
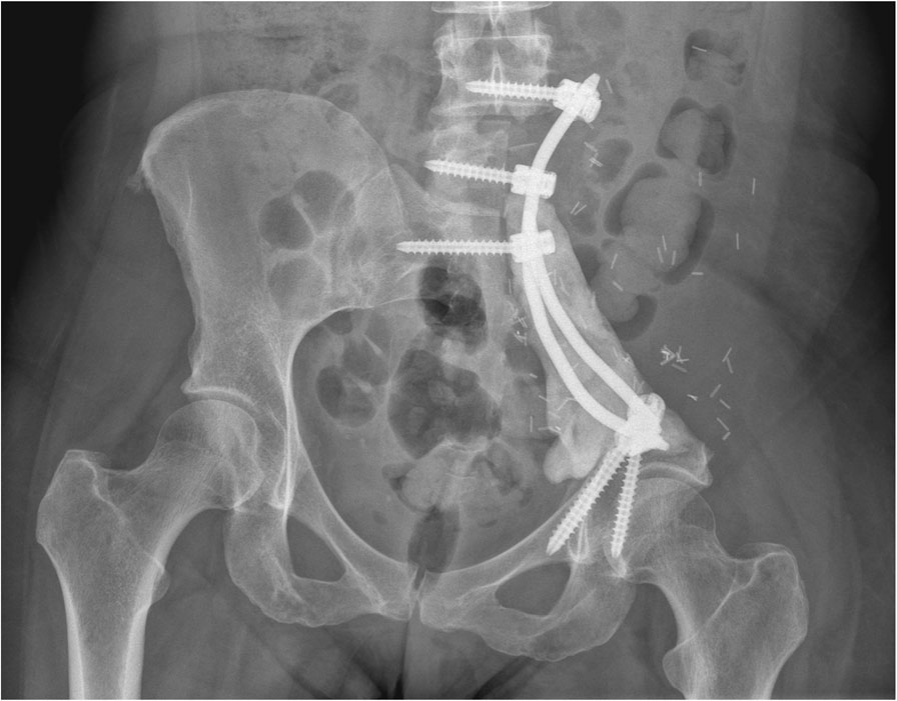
Plain radiograph of a pelvis a.p. after type 1c resection including partial resection of the fifth lumbar vertebrae and reconstruction with a polyaxial screw-rod-system augmented by a bone cement sheath

Reconstruction of resulting pelvic bone defects largely depended on the type of resection performed and surgeon’s preference. In general, limb-salvaging so-called internal intraarticular pelvic resections or hemipelvectomies were commonly reconstructed by hip transposition (Fig. 2), which describes the process of transposing the femoral head and approximating it to the proximal osteotomy level. It was there embedded in a newly formed joint capsule consisting of remaining iliopsoas and gluteus muscles and sometimes augmented using attachment tubes or bone anchors. It resulted in limb length discrepancies and lead to permanent functional disability while retaining the ability to walk (sometimes with crutches). Extraarticular resections were similarly reconstructed (Fig. 3), replacing the proximal femur with a prosthetic implant. Large megaendoprosthetic pelvic implants have been proposed in the past, but high failure and infection rates caused a preference in performing reconstructions that manage without large endoprosthetic surfaces at this department. Pelvic resections of the pubic and ischial bone, which did not or only marginally affected the acetabulum, were reconstructed by soft tissue rearrangement and joint capsule reconstruction only, as weight bearing was not impaired severely compared with other resection types (Fig. 4). Acetabulum-retaining resections of the posterior pelvic ring (i.e., type P1c) were reconstructed using poly-axial screw rod reconstructions augmented by a bone cement sheath (Fig. 5). Alternative biological reconstructions were autologous iliac wing, autologous fibula, or allograft compound osteosyntheses. The main goal of defect reconstruction was improving primary stability and avoiding long-term shortening of the limb caused by approximation of osteotomy levels if those defects were left without reconstruction.

Pelvic resection specimens were analyzed histologically with regard to confirmation of histological diagnosis of ES, evaluation of tumor margins, and response to neoadjuvant chemotherapy according to the classification proposed by Salzer Kuntschik [37].

Postoperative (adjuvant) radiation therapy (45–54 Gray (Gy)) was recommended by the ITB for positive as well as clear but close resection margins, poor response to neoadjuvant chemotherapy, and large initial tumor volumes. Preoperative (neoadjuvant) radiation therapy (54 Gy) was considered if resection margins were expected to be close or clinical response to chemotherapy poor. Definitive radiation therapy (54–64 Gy) was recommended when pelvic tumor resection would have been mutilating or clear resection margins could not be achieved.

Brachytherapy, also called internal radiation therapy, was recommended in 1994 (*n* = 1) and between 2001 and 2004 (*n* = 9). For brachytherapy, a radioactive source was placed directly adjacent to the tumor bed after pelvic tumor resection, enabling delivery of a high dose of radiation without first passing through non-target tissues. It necessitated a second operation to explant the radioactive source and was discontinued after 2004.

Statistical evaluation was performed using the SPSS Statistics 25 software. The Kaplan-Meier estimation was used to analyze survival and univariate analysis to analyze and compare single influencing parameters. Statistical analysis only included patients with primary pelvic ES and complete follow-up. A *p* value of < 0.05 was accepted as statistically significant.

### Patient characteristics

An overview of relevant patient- and treatment-related characteristics is presented in Table 1. The mean age in this patient cohort was 18.1 years, and patients were generally healthy, presenting only rarely with relevant pre-existing conditions. Among these, arterial hypertension (*n* = 2), pelvic deep vein thrombosis (DVT) with (*n* = 2) and without (*n* = 1) pulmonary embolism, diabetes (*n* = 2), obesity (*n* = 2), and a history of malignant ovarian germ cell tumor (*n* = 1) and Hodgkin’s disease (*n* = 1) were observed.

Tumor size was characterized by centimeter in size in the longest diameter and tumor volume (when known). The mean tumor size was 8.8 cm in the longest diameter. Thus, the patient cohort was divided into two groups (< 9 cm, > 9 cm) for further analysis. Tumor volume (</> 200 ml) was documented whenever information was available.

Primary pelvic resections were performed in ninety-six patients (93.3%) and as secondary procedures after primary intralesional procedures elsewhere in seven patients (6.7%).

All but three patients were indicated to undergo pelvic tumor resection for tumors resectable with clear margins and a curative treatment intent. Exceptions were made for two pelvic tumor resections with planned-positive margins. One patient had ipsi- and contralateral sacral involvement and was clinically expected and later confirmed to have a good response to chemotherapy (no residual vital tumor cells). Positive (R1) margins were tolerated in this patient as resection with clear margins would have compromised motor function of both limbs, and the patient was recommended to undergo adjuvant radiation therapy. The other patient actually had a resection with clear margins as determined by the pathologist. Nevertheless, the resection was considered contaminated and adjuvant radiation therapy recommended on grounds of excessive lavage of the pelvis for a suspected diagnosis of osteomyelitis at the primary care clinic. Only one patient was treated with a palliative treatment intent by hindquarter amputation to relieve pain and improve quality of life.

All but this one patient, who underwent hindquarter amputation, had limb-salvaging tumor resections (*n* = 103). Hip transposition was the most common reconstruction technique after pelvic tumor resection including the acetabular socket (*n* = 48), followed by compound osteosynthetic reconstructions using a polyaxial screw-rod system and polymethyle methacrylate (PMMA) sheath, autologous fibula, or allograft (*n* = 32) for acetabulum-sparing resections of the upper posterior pelvis. Nineteen patients had soft tissue reconstructions for isolated pP2 and P3 resections. Patients with extraarticular resections (*n* = 13) were reconstructed using proximal femur megaendoprostheses (*n* = 9), a spacer (*n* = 2), a pelvic megaendoprosthetic implant, and flail hip in one case each. The mean operation time was 255.75 min (range 48–525 min; *n* = 78). Information on the blood loss was available in 77 patients who received a mean of 4.9 erythrocyte concentrates (EC) (range 0–30 EC) and 4.4 fresh frozen plasmas (FFP) (range 0–27, *n* = 71). After operation, adjuvant chemotherapy was continued after a mean time of 20 days (range 10–43 days; *n* = 53) after operation.

Pelvic radiation therapy was administered in 77.9% (*n* = 81/104) of patients. The mean pelvic radiation dose administered was 45.7 Gy (range 12.6–64.4 Gy). Eleven radiation treatments were specified as “other” (Table 1) and follow in decreasing order: brachytherapy and postoperative radiation, *n* = 5; brachytherapy only, *n* = 3; preoperative radiation and brachytherapy, *n* = 1; pre- and postoperative radiation therapy, *n* = 1; and preoperative hyperthermia and postoperative radiation therapy, *n* = 1.

## Results

**Fig. 6:**
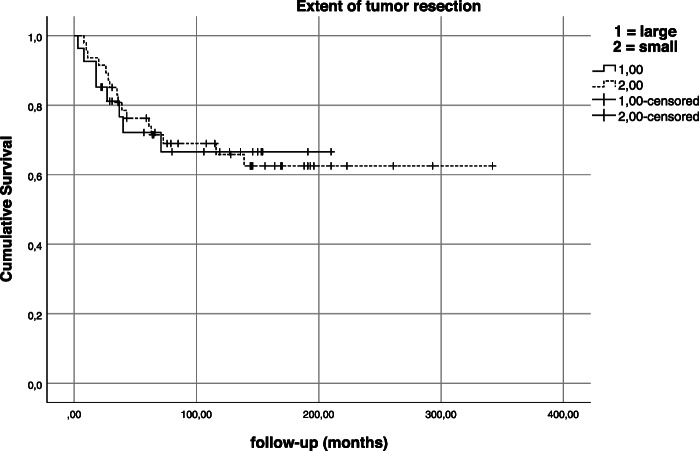
Kaplan-Meier estimation—impact of extent of tumor resection on survival

Pelvic resections types were subdivided into partial/subtotal hemipelvic resections (extending to both sides of the acetabulum) and small pelvic resections (involving the anterior or posterior pelvic ring only). However, partial or near total resection of the hemipelvis did not have an impact on local recurrence (*p* = 0.795) or overall survival (*p* = 0.602; Fig. 6).

In this patient cohort, we did not observe any deaths caused by pelvic tumor resection during operation or reconvalescence. We did however observe both major as well as minor intraoperative, perioperative, and long-term complications caused as a direct result of pelvic tumor resection, chemotherapy, and radiation therapy (see Table 2). Proximity to neurovascular structures as well as pelvic organs lead to injury of the lumbosacral plexus, meningeal membranes, internal or external iliac vessels, bladder, urethra, ureter, vagina, and vulva a total of 40 times. Those major complications did not all occur separately but often as combination injuries because of unexpected adherence of the tumor to its surroundings, necessitating intraoperative consultation with medical specialists in urology, gynecology, or neurosurgery. However, intraoperative injury of vessels and pelvic organs ultimately healed. Loss of motor function of the affected limb caused by irritation of the nerve roots during preparation was often transient. Permanent loss of function was likely caused in part by immobility and bed rest with compression of the nerve roots of the lumbosacral plexus by the approximated femur after hip transposition (most commonly causing peroneal or sciatic palsy) rather than injury during operation alone.

**Table 2:** Surgical, chemotherapy-, and radiotherapy-associated complications

**Surgical complications**				**Chemotherapy-associated complications**							
**Intraoperative**		**Urogenital/intestinal intra- and perioperative**		**Neoplastic**			*n*	**Neurological**			*n*
*Major*	*n*	*Minor*	*n*	Tumor progression under chemo			1	Polyneuropathy			8
Lumbosacral plexus injury (manipulation)	24	Urinary tract infection	10	**Cardiovascular**			*n*	Epileptic seizure			4
		Pyelonephritis	1					Psychosis			2
Iliac vessel injury	3	Testicular varicocele	1	Cardiomyopathy			4	Temporary character change			1
Cerebrospinal fluid leak	2	Iatrogenic undescended testis	1	Long QT syndrome			2	Reduced vigilance			1
Implant malposition (screw)	1	**Long-term**		DVT (associated w/ Port-a-cath)			2	Myoclonic twitches			1
*Minor*	*n*	Limb shortening	49	Sinus bradycardia			1	Impaired coordination			1
Allergic reaction	1	Chronic pain	26	Arterial hypertension			1	Encephalopathy			1
**Perioperative**		Femoral head necrosis	21	Ventricular tachycardia			1				
*Major*	*n*	Secondary scoliotic deformity	17	SVT and AVNRT			1	**Acoustic**			*n*
Diarrhea, enteritis, colitis	14	Implant failure (screw)	14	**Nephrological**			*n*	High-frequency hearing loss			1
Sepsis/SIRS	1/1	Lymphedema, erysipelas	14	Chronic renal failure			11	Acute hearing loss			1
Cerebrospinal fluid fistula	1	Inactivity-induced osteopenia	12	Tubular nephropathy			7				
Spinal hematoma (residual permanent neurological deficit)	1	Talipes equinus	11	Fanconi syndrome			6	**Infectious/allergic**			*n*
		Coxarthritis	9								
Ischemia lower limb	1	Fracture	8	**Endocrinological**			*n*	Pneumonia			7
*Minor*	*n*	Pseudarthrosis	5	Secondary amenorrhea			8	Sepsis			6
DVT	3	Dislocation	4	Hypergonadotropic hypogonadism			8	Infection port-a-cath			4
Allergic reaction	1	THR	3	Ovarian insufficiency			1	Septic multi-organ failure			1
**Urogenital/intestinal intra- and perioperative**		Depressive episodes	2	Hypergonadotropic azoospermia			1	Transfusion-associated allergic reaction			14
		Scar hernia	2								
*Major*	*n*	Suicide attempt	1								
Urinary incontinence	7	Stasis eczema	1								
Ureter stenosis	5	Hyperlordosis	1	**Radiotherapy-associated complications**							
Urethra injury	4	Implant loosening	1								*n*
Bladder injury	3			Radiation-induced dermatitis			5	Radiation-induced osteosarcoma			1
Vagina injury	3	**WHD and DWI**									
Vulva injury	1	Total	32								
Urosepsis	2	Early	18								
Iatrogenic urinoma	1	Late	14								
Fistula (bladder/abdominal wall)	1	Conservative antibiotics	3								
Fecal incontinence	1	Operative revision	29								

While perioperative complications such as DVT or urinary tract infection were considered minor, the most frequent major complication observed was superficial wound healing disorder (WHD) and deep wound infection (DWI) affecting 32 patients (30.8%; *n* = 32/104). Superficial WHD was defined as the flap or skin necrosis and diagnosed in 18 patients (56.3%, *n* = 18/32; 17.3%, *n* = 18/104). Among these, three patients were treated with a course of antibiotics and healed without surgical intervention; the remaining 15 patients either progressed to DWI or needed revision operations for superficial WHD. DWI was defined as bacterial infection of deep hematoma or fatty tissue and muscle necrosis. It occurred within the first 2 weeks after pelvic resection and was diagnosed by fever, elevated C-reactive protein levels (CRP), or wound seepage. Over the years, DWI also occurred either due to hematogenous spread of bacteria or reactivation of dormant low-grade infection in 14 patients (43.7%, *n* = 14/32; 13.5%, *n* = 14/104) who did not show symptoms of WHD or DWI during primary wound healing. It was diagnosed only when primary wound healing had concluded with normalization of CRP levels and removal of suture material, at least 4 weeks or longer after pelvic tumor resection. DWI showed a tendency towards recurrent infection of the operation field. The mean rate of DWI per person was 1.5 (range 1–4). Of 32 patients affected by superficial WHD and both early and/or late DWI, 29 patients (90.6%) required surgical revision operations. A mean of three operations per patient were performed (range 1–10). Operative and antibiotic treatments were successful to a degree that hindquarter amputation was avoided in all 32 affected patients.

Other long-term complications, such as limb length discrepancies causing physical disability were observed in 49 patients (47.1%). The mean shortening was 6.8 cm (range 1–20 cm). Twelve patients in this collective underwent limb lengthening, adapting or function enhancing procedures: distraction osteogenesis (DO) with intramedullary lengthening nail (*n* = 5), DO with Ilizarov fixator (*n* = 3), and temporary epiphyseodesis around the knee (*n* = 3). One patient had posterior tibial tendon transfer for postoperative peroneal palsy caused by injury or irritation of the lumbosacral plexus during or after pelvic tumor resection.

Among oncological complications, local recurrence occurred in eight patients (7.7%) after a mean time of 21.5 months (range 2–39 months) after pelvic resection. Risk factors for developing local recurrence, such as distant metastasis at operation (*n* = 4), poor response to chemotherapy (*n* = 3), primary multifocal disease (*n* = 2), prior intralesional resection at a primary care hospital (*n* = 2), disease progress despite neoadjuvant chemotherapy (*n* = 1), relapse after definitive radiation therapy (*n* = 1), and contaminated resection margin (*n* = 1), were observed in seven patients. Only one patient remains alive without evidence of disease at 99 months after pelvic resection and 63 months after re-resection for local recurrence. The other seven patients ultimately died of their disease at a mean follow-up of 32.1 months. Local recurrence proved to be negative predictive for overall survival in univariate analysis (*p* = 0.001).

**Fig. 7:**
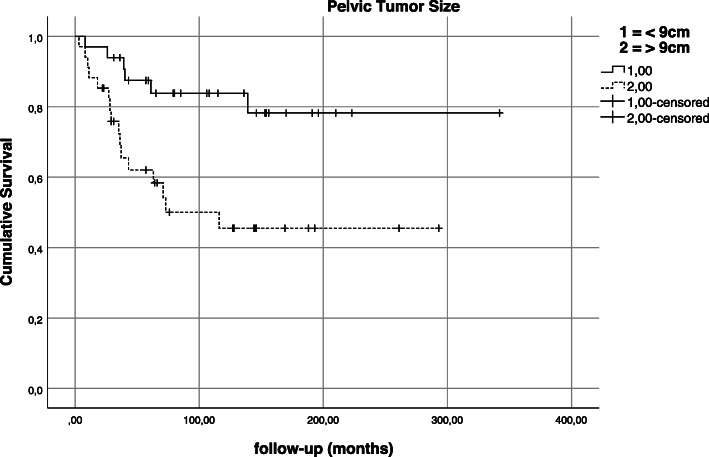
Kaplan-Meier estimation—impact of tumor size on survival

Tumor size, in this patient cohort, did not have an impact on local recurrence (*p* = 0.626) or metastatic status at operation (*p* = 0.421). Larger tumor size, however, was observed to be negative predictive for overall survival (*p* = 0.005) in univariate analysis (Fig. 7).

**Fig. 8:**
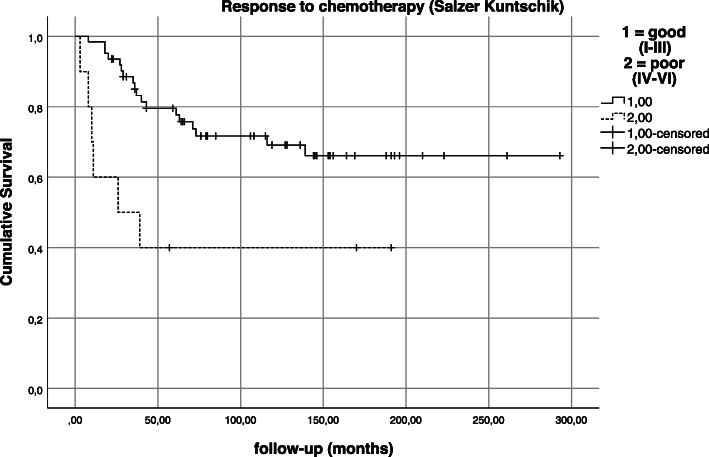
Kaplan-Meier estimation—impact of response to neoadjuvant chemotherapy on survival

Response to neoadjuvant chemotherapy was good (< 10% vital tumor) in 78.8% (*n* = 82/104), poor (grades IV–VI) in 18.3% (*n* = 19/104) and not available in 2.9% (*n* = 3/104) of cases (Fig. 8). Response to chemotherapy was observed to have a statistically significant impact on overall survival in this patient collective (*p* = 0.036).

**Fig. 9:**
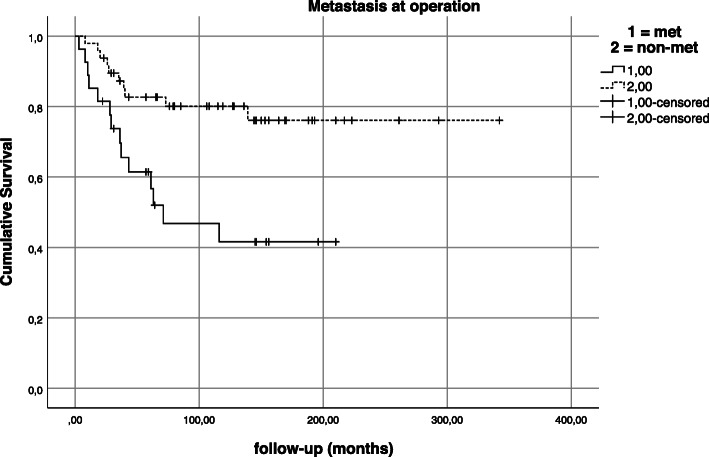
Kaplan-Meier estimation—impact of metastatic status at operation on survival

**Fig. 10:**
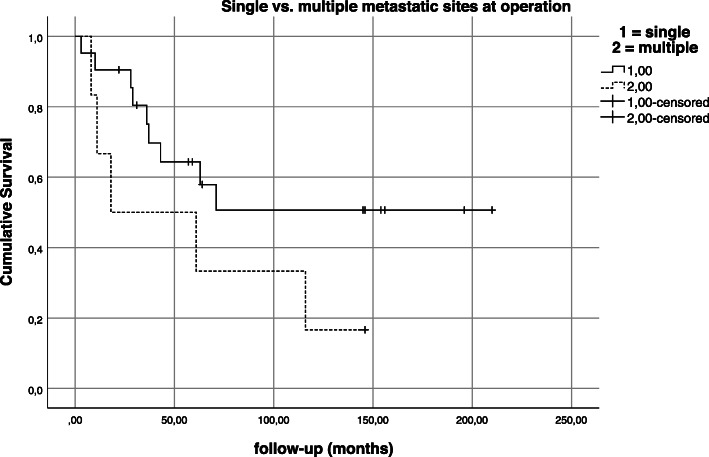
Kaplan-Meier estimation—impact of number of metastatic sites on survival

Univariate analysis also yielded that status of distant metastases at the time of operation proved to be an important predictive factor for overall survival (*p* = 0.003; Fig. 9). At the time of diagnosis/operation, distant metastases were absent in 56.1% (*n* = 46) and diagnosed in 35.4% (*n* = 29) of patients. 15.9% (*n* = 13) developed a distant metastasis after a mean time of 34.6 months after pelvic resection. Permanent absence of distant metastases was observed in 33 patients (40.2%). Twenty-two patients (75.9%) with initial distant metastases had a single-metastatic site (i.e., only pulmonary or lymphatic metastasis) while seven patients (24.1%) suffered from two or more metastatic sites (combination of at least two different organs/sites). And while the number of distant metastatic sites at the time of operation did not have a statistically significant impact on survival (*p* = 0.130; Fig. 10), the cumulative 5-year and 10-year survival for patients with a single distant metastatic site was 64.3% and 50.7% compared to 50% and 16.7% in patients with multiple distant metastatic sites. In comparison, the overall survival for patients with distant metastases regardless of their number of sites at the time of operation was 61.4% and 41.6% at 5 and 10 years compared to 82.7% and 80.1% for patients without distant metastases at the time of operation.

**Fig. 11:**
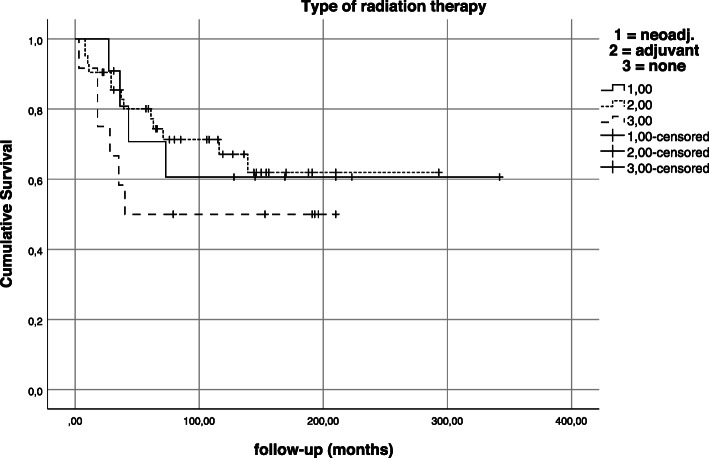
Kaplan-Meier estimation—impact of type of radiation therapy on survival

Patients treated with neoadjuvant and adjuvant radiation therapy had similar outcomes with regard to overall survival and were superior to patients who did not receive radiation treatment in this patient cohort. However, these findings were not statistically significant (*p* = 0.424; Fig. 11).

When collection of follow-up data was completed in October 2016, 56 patients (53.8%) remained alive at a mean follow-up of 145.5 months (range 22–340 months). Thirty-seven patients (35.6%) had died of disease after a mean time of 78.9 months (range 1–171) after pelvic resection. Of these, patients with initial metastatic disease (*n* = 24) and metastatic disease after pelvic resection (*n* = 11) died after a mean time of 31.9 (1–116) and 52.8 (8–171) months. Four patients (3.8%) had died of other causes (secondary malignancy, *n* = 3; multi-organ failure (chemotherapy-induced), *n* = 1) after a mean time of 34.8 months (range 3–64 months). Seven patients (6.7%) were lost to final follow-up.

## Discussion

This study presents a patient cohort of one-hundred four patients treated for pelvic ES by tumor resection in all and additional radiation therapy in 77.9% of cases at a single supra-regional center between 1988 and 2014. Despite large tumor sizes (41.3% diagnosed with tumors > 9 cm in the longest diameter), clear surgical margins were achieved in 94.2% and response to neoadjuvant chemotherapy was good with less than 10% viable tumor in 78.9% of cases. Non-metastatic patients at the time of diagnosis/operation had a cumulative 5- and 10-year overall survival of 82.7% and 80.1%, respectively.

Ahmed et al. presented their findings regarding local control and survival for pelvic ES patients treated from 1990–2012 (*n* = 48). They included eight patients treated by tumor resection alone (localized, *n* = 6; metastatic (lung-only), *n* = 2), thirty-one patients treated by radiation therapy only (localized, *n* = 15; metastatic, *n* = 16), and eight patients treated by operation and radiation therapy (metastatic, *n* = 6; initial diagnostic resection at primary care center before chemotherapy, *n* = 2). One patient did not receive local treatment. Their 5-year overall survival rates for all, and localized and metastatic patients were 50%, 73%, and 30% [35].

Krasin et al. published their analysis of local outcome and prognostic factors for patients with localized ES of both favorable (extremity, head, neck; *n* = 20) and unfavorable (chest, abdomen, pelvis; *n* = 13) tumor sites treated with definitive surgery in 2005. The 5- and 10-year overall survival rates were 84.5% and 75.8%, the cumulative incidence of local recurrence was 12.5% [38].

Rodríguez-Galindo et al. report their analysis of prognostic factors in ES (*n* = 220; 1979–2004) in 2007 and subdivide outcomes into four groups: favorable (age < 14 years with localized, non-pelvic tumors), intermediate risk (localized, age > 14 years, or pelvic tumors), unfavorable-pulmonary (isolated lung metastases), and unfavorable-extrapulmonary (extrapulmonary metastases). The 5-year OS estimates for these groups were 88.1%, 64.9%, 53.8%, and 27.2% [39].

The OS rates reported for non-metastatic pelvic ES in this study are higher compared with Ahmed et al. who investigated both non- and metastatic pelvic tumors but treated by radiation only in 64.6% and used combined local treatment approaches in eight unfavorable constellations only (16.7%) [35]. This study’s findings resemble OS rates published by Krasin et al. who report OS rates for a mixed patient cohort, including both pelvic and non-pelvic primary tumor sites. In comparison with Rodríguez-Galindo et al., this study’s OS rates for non-metastatic pelvic ES rank between results achieved in their favorable and intermediate-risk groups. Therefore, the pelvic ES OS estimates published in this study compete with those achieved for non-pelvic ES tumors.

A possible explanation for these results, which are in contrast with the dismal outcomes usually reported for pelvic ES in literature, may be the high rate of combined local treatment in this patient cohort despite a high rate of clear resection margins and good clinical and histopathological response to neoadjuvant chemotherapy. The reasoning behind indicating adjuvant radiation treatment for this patient cohort in ITB meetings were the incidence of generally large tumors and a certain doubt whether wide resections of pelvic tumors were ever truly possible due to a close proximity with adjacent organs and neurovascular structures. In addition, uncertainty remained whether the pre-chemotherapy tumor dimensions were entirely captured within the resection specimen despite clear resection margins.

Reviewing existent pelvic ES literature [8, 10, 25–27, 29, 30, 40–42] with this subgroup’s parameters in mind, and allowing for differences in surgical technique and time of treatment, Frassica et al. already reported a 5-year OS rate of 75% for eight localized pelvic ES patients who were treated by a combined local approach in 1993 [25]. In 2008, Indelicato et al. also reported a 15-year actuarial cause specific survival of 76% for combined treatment approaches in their study [29].

The rate and type of surgical complications observed in this study were comparable with other reports in literature [1, 5, 43]. Angelini et al. published a rate of 20% for deep wound infections, which occurred more frequently in reconstructed patients (26%) compared with patients without reconstruction (15%) [44]. These findings compare with an infection rate of 30.8% in this collective. Since infection is more common in reconstructed patients, the use of endoprosthetic or large foreign-body materials needs to be weighed against its functional long-term benefits. In this regard, Puri et al. published acceptable function scores for unreconstructed patients (*n* = 13/26) who had acetabulum-retaining operations. Reported musculoskeletal tumor society scores ranged from 23 to 29 [42]. As a fairly young and healthy patient cohort was examined in this study, we did not reach infection rates, which are reported to be as high as 61.7% [45] in elderly patients with comorbidities, and did not find operation-associated deaths. Complications did not lead to secondary hindquarter amputations either. Kollender et al. confirm these findings in their 2000 study of twenty-seven patients who underwent internal hemipelvectomy for bone sarcoma (*n* = 24/17 ES). They report no need for reconstruction in 44.4%, infections in 14.8%, and no need for secondary hindquarter amputation or operation-associated deaths in their collective. Their local reccurence rate was 22% (*n* = 24/27 pelvic ES) [46]. The local recurrence in this study was relatively low with 7.7% compared with other reports in literature [38]. However, as Foulon et al. report that radiotherapy appears to improve local control even in patients with complete tumor necrosis after neoadjuvant chemotherapy [31], the high incidence of radiation therapy in this collective may also positively affect the high rate of local control reported by combined local treatment in this study.

An acceptable complication rate for pelvic tumor resection and radiotherapy in this study and OS rates for non-metastatic pelvic primaries approaching survival rates published for extremity locations, appear to warrant a combined treatment approach in this collective’s non-metastasized subgroup. Andreou et al. whose analysis of data from the Euro-Ewing 1999 trial confirms that in a subgroup analysis of pelvic ES patients with wide surgical margins and a good histologic response to induction treatment, combined local treatment was associated with a higher overall survival probability (87% vs. 51% at 5 years), compared with surgery alone [33]. Current UK and ESMO (European Society for Medical Oncology) guidelines reinforce these findings and recommend complete surgery, where feasible, as a local treatment. Whenever infeasible and tumors cannot be resected with clear margins, definite radiation therapy should be applied. Incomplete resections should be avoided, as incomplete surgery followed by radiation therapy did not prove to be superior to radiation therapy alone [47, 48]. The current Euro-Ewing-2012 guidelines recommend postoperative radiotherapy for positive margins with microscopic residual disease (R1-2) (unless re-resection is possible with clear margins), if all tissues involved by the pre-chemotherapy tumor volume have not been excised (even if resection margins are negative), after displaced pathological fracture at primary tumor site, and in certain tumor sites where local control is judged to be more difficult to achieve (i.e. pelvis) [48].

The single- and multiple-metastatic site OS estimates in this patient cohort are 64.3% versus 50.7% at 5 years and 50% versus 16.7% at 10 years. Ahmed et al. who report 5-year OS and event-free survival (EFS) rates of 30% and 18% for metastatic patients confirm these findings. Studies by Haeusler et al. (2010) report that local therapy of involved sites is important even for patients with primary, disseminated, multifocal ES (PDMES) [49]. In addition, Ladenstein et al. (2010) found that PDMES patients might survive with intensive multimodal therapy and local therapy consisting of surgery and/or radiation therapy [50].

The significance of pelvic tumor resection in pelvic ES primaries remains unknown, and counseling patients with metastatic pelvic ES remains a challenge. Long breaks from multi-agent chemotherapy caused by wound infections likely lead to distant tumor progression, and the overall risks and complications of pelvic tumor resection with resultant hospitalization and physical disability have to be weighed against quality of life aspects. Future investigations determining this patient collective’s benefit of undergoing primary tumor resection are necessary.

Published outcomes for photon beam radiation may be surpassed by proton beam radiation in the future. Pelvic ES already is a main field of application for proton beam therapy in both non- and metastatic pelvic ES for both definitive as well as adjuvant radiation treatment. Uezono et al. recently published their results of thirty-five patients treated by definitive (*n* = 26), preoperative (*n* = 7), and postoperative radiation (*n* = 2) therapy for pelvic ES. They reported a 3-year overall survival, progression-free survival, and local control rates of 83%, 64%, and 92%. Depending on pending long-term results achieved by proton beam radiation with regard to survival, local control, complications, and long-term effects in pelvic ES patients, it may well gain importance and improve treatment of pelvic ES [51].

## Conclusion

This study supports a combined local treatment approach for non-metastatic pelvic ES. In addition, by achieving clear resection margins and considering reported short- and long-term complications, pelvic resections are a feasible treatment modality for ES with pelvic primary. Single-metastatic site in this study and solitary pulmonal metastases as reported in literature have outcomes that are more favorable compared with multiple-site and extrapulmonal metastases. Indication for pelvic tumor resection should be considered bearing these findings in mind. As of now, recommendations for local treatment have to be made on a case-by-case basis. Preliminary results reported for proton beam therapy for pelvic ES in literature are promising and may have an impact for improving treatment strategies in the future.

### Limitations

We acknowledge the shortcomings of retrospective study design. The lack of a control group limits the significance of our findings, as we are unable to compare different treatment subgroups. In addition, there is an inherent risk of selection bias of patients who were recommended to undergo pelvic tumor resection, possibly affecting the outcomes observed in this study.

## Data Availability

The datasets used and analyzed during the current study are available from the corresponding author on reasonable request.

## Abbreviations

ES: Ewing sarcoma
UK: United Kingdom
GPOH: German Society for Pediatric Oncology and Hematology
Gy: Gray
DVT: Deep vein thrombosis
PMMA: Polymethyl methacrylate
EC: Erythrocyte concentrate
FFP: Fresh-frozen plasma
WHD: Wound healing disorder
DWI: Deep wound infection
DO: Distraction osteogenesis
ESMO: European Society for Medical Oncology
EFS: Event-free survival
PDMES: Primary disseminated multifocal Ewing sarcoma
SIRS: Systemic inflammatory response syndrome
THR: Total hip replacement
w/: With
SVT: Supraventricular tachycardia
AVNRT: Atrioventricular nodal reentry tachycardia
